# Bioassay-Guided Isolation of Cytotoxic Cycloartane Triterpenoid Glycosides from the Traditionally Used Medicinal Plant *Leea indica*

**DOI:** 10.1155/2012/164689

**Published:** 2011-11-29

**Authors:** Yau Hsiung Wong, Habsah Abdul Kadir, Sui Kiong Ling

**Affiliations:** ^1^Biomolecular Research Group, Biochemistry Division, Institute of Biological Sciences, Faculty of Science, University of Malaya, 50603 Kuala Lumpur, Malaysia; ^2^Division of natural products, Forest Research Institute of Malaysia (FRIM), 52109 Kepong, Malaysia

## Abstract

*Leea indica* is a medicinal plant used traditionally to cure cancer. In this study, the cytotoxic compounds of *L. indica* were isolated using bioassay-guided approach. Two cycloartane triterpenoid glycosides, mollic acid arabinoside (MAA) and mollic acid xyloside (MAX), were firstly isolated from *L. indica*. They inhibited the growth of Ca Ski cervical cancer cells with IC_50_ of 19.21 **μ**M (MAA) and 33.33 **μ**M (MAX). MRC5 normal cell line was used to calculate selectivity index. MAA and MAX were about 8 and 4 times more cytotoxic to Ca Ski cells compared to MRC5. The cytotoxicity of MAA was characterized by both cytostatic and cytocidal effects. MAA decreased the expression of proliferative cell nuclear antigen, increased sub-G1 cells, and arrested cells in S and G2/M phases. This study provides the evidence for the ethnomedicinal use of *L. indica* and paves the way for future mechanism studies on the anticancer effects of MAA.

## 1. Introduction

Plants provide us with broad spectrum of biologically active compounds that have potential therapeutic effects on a myriad of diseases. *Leea indica* (Burm. f.) Merrill is a traditional Chinese medicine which belongs to the Leeaceae family. It is a perennial shrub which is widely grown in tropical and subtropical countries such as Malaysia, China, India, and Thailand. The leaves and roots of *L. indica* are used to treat diabetes, cardiac diseases, and various ailments such as fever, headache, dizziness, soreness, eczema, sprain, leprosy, bone fracture, body pain, muscle spasm, diarrhea, and dysentery [1–7]. In view of that, some phytochemical studies have been conducted [8–11]. For biological studies, antimicrobial, antioxidant, antiinflammatory, hypo-glycemic, and phosphodiesterase inhibitory activities have been reported in *L. indica* [10–16]. In Leeaceae family, *L. guineense *and *L. macrophylla *were ethnomedicinally used to treat cancer [17, 18]. For *L. indica*, it is used as an ingredient in the preparation to treat leucorrhea, intestinal cancer, and uterus cancer [19]. The leaf decoction is consumed by women during pregnancy and delivery for birth control or to treat obstetric diseases and body pain [20, 21]. In addition, the dried leaves are consumed as a tea beverage and are believed to be effective against cancer [22].

In our previous cytotoxicity screening, the crude ethanol extract and fractions (ethyl acetate, hexane, and water) were found to inhibit the growth of Ca Ski cervical cancer cell line [23]. This provides the evidence for the use of *L. indica* as folkloric treatment of cancer. In the present study, we reported the further progress whereby the active fraction (ethyl acetate) of *L. indica* was subjected to bioassay-guided approach in order to isolate the cytotoxic compounds from *L. indica.*

## 2. Methods

### 2.1. Plant Sample Collection, Identification and Deposition of Voucher Specimen

From the previous report [23], the fresh leaves of *L. indica* were collected, authenticated, extracted, and fractionated. A voucher specimen (47365) was deposited at the herbarium of the Institute of Biological Sciences, Faculty of Science, University of Malaya, Kuala Lumpur, Malaysia.

### 2.2. Bioassay-Guided Isolation of Active Constituents from the Ethyl Acetate Fraction of *L. indica*

The active ethyl acetate fraction (50 g) was dissolved in MeOH and loaded into Diaion HP-20 SS (Supelco, Bellefonte) column, eluted using a gradient solvent system of 40% MeOH and 60% H_2_O with 10% MeOH increment. Thin layer chromatography (TLC) analysis was performed on precoated silica gel 60 F_254_ plates (0.2 mm thick, Merck), and spots were detected by UV illumination after spraying with 10% H_2_SO_4_ followed by heating. Based on the TLC profiles, a total of nine combined fractions (designated F1–F9) were pooled together. MTT assay was performed on each fraction. The active F8 was further subjected to silica gel (200–400 mesh, Merck) column chromatography. The mobile phase consisted of CHCl_3_ : MeOH : H_2_O (C : M : H, v/v). The initial solvent composition was 100% C, and then it was changed to C : M (9.5 : 0.5), followed by C : M : H (9 : 1 : 0.1), C : M : H (8.5 : 1.5 : 0.1), C : M : H (8 : 2 : 0.2), C : M : H (7 : 3 : 0.5), C : M : H (6.5 : 3.5 : 0.5), C : M : H (6 : 4 : 1), and finally to 100% M. A total of six fractions (F81–F86) were obtained. The active F83 was further fractionated again on silica gel 60 column using C : M : H system. The initial solvent was 100% C, and then it was changed to C : M : H (9 : 1 : 0.1), followed by C : M : H (8.5 : 1.5 : 0.1), C : M : H (8 : 2 : 0.2), C : M : H (7 : 3 : 0.5), and finally to 100% M. Another six fractions (F831–F836) were obtained. The active F835 was further purified by prep-TLC (silica gel 60 F_254_ glass plates, size 20 cm × 20 cm, Merck) using C : M : H (7 : 3 : 0.5) as solvent system and yielded compounds **1** (55.9 mg) and **2 **(26.6 mg).

### 2.3. Elucidation of Structural Compound

For structural elucidation purposes, the compounds were subjected to instrumental analysis at the Forest Research Institute Malaysia (FRIM), Selangor, Malaysia. Structures were elucidated mainly using nuclear magnetic resonance (NMR) techniques and Liquid Chromatography/Mass Spectrometry (LC/MS). The compounds were dissolved in pyridine-d5 solution. The ^1^H, ^13^C, and distortionless polarization transfer (DEPT-135) NMR spectra were recorded on a Bruker DRX 300 NMR spectrometer. The internal reference standard used was tetramethylsilane (TMS). LC-MS analysis was performed using LTQ Orbitrap mass spectrometer (Thermo Fisher Scientific) fitted with an electrospray interface.

### 2.4. Cell Culture

The human cervical epidermoid carcinoma cell line (Ca Ski, ATCC number CRL-1550) and human fibroblast cell line (MRC 5, ATCC number CCL-171) were purchased from the American Type Culture Collection (ATCC, USA). Ca Ski cells were maintained in RPMI 1640 Medium (Sigma) and MRC 5 cells in EMEM (Eagle Minimum Essential Medium) (Sigma). The media were supplemented with 10% (v/v) heat-inactivated fetal bovine serum (PAA Lab, Austria), 100 *μ*g/mL streptomycin and 100 unit/mL penicillin (PAA Lab, Austria), and 50 *μ*g/mL amphotericin B (PAA Lab, Austria). The media were filter-sterilized using a 0.22 *μ*m filter membrane (Minisart, Sartorius stedim). The cells were cultured in 5% CO_2_ incubator at 37°C in a humidified atmosphere.

### 2.5. MTT Assay

MTT assay was modified from Mossmann and used to evaluate the cytotoxic effects of each fraction and compounds. MTT assay is widely used to assess the viability and/or the metabolic state of the cells based on mitochondrial respiratory activity [24]. A total of 5 × 10^3^ cells were seeded into each well of 96-well plates and allowed to adhere for 24 h. After 24 h, the cells were treated with the fractions or compounds in the final concentrations ranging from 3.125 to 100 *μ*g/mL. Each concentration was performed in triplicate well. Control cells were treated with vehicle DMSO to get the final concentration of 0.5% v/v. The cells were then incubated for 72 h. After 72 h exposure period, MTT (5 mg/mL) was added and further incubated for 4 h at 37°C. The medium was then aspirated and the crystals formed were dissolved in 150 *μ*L DMSO. The absorbance was measured at 570 nm against the reference wavelength of 650 nm. The percentage of viability was calculated based on the formula: Viability (%) = (absorbance of treated cells/absorbance of control cells) × 100%. The IC_50_ (concentration that reduces cell viability to 50%) was derived from the dose-response curves.

MRC 5 cell line was used as a normal cell model for the calculation of selectivity index (SI). SI value was calculated by dividing the IC_50_ value of the MRC 5 cell line with the IC_50_ value of Ca Ski cell line [25, 26].

In order to determine whether the cytotoxicity is cytostatic or cytocidal, a recovery assay was conducted whereby after the 72 h incubation (exposure period), the medium containing the compound was removed, washed with medium, and replaced with medium alone for a recovery period of 72 h followed by addition of MTT and measurement as described earlier. A sample is showing cytostatic effect when the IC_50_ in the recovery assay was higher than that of the exposure assay. Whereas for cytocidal effect, the IC_50_ obtained in the recovery assay is similar to that shown by the exposure assay [27, 28].

### 2.6. Trypan Blue Exclusion (TBE) Assay and Observation of Cell Morphological Changes

Ca Ski cells were cultured at a density of 1 × 10^6^ cells/mL in 60 mm culture dishes. After 24 h of attachment, the cells were treated without or with 60 *μ*M of MAA for different time periods. After 6–72 h, the cells were harvested, washed with medium, and the cell pellets were resuspended in medium. After incubation in 0.4% trypan blue for 5 min, viable cells were counted using a hemocytometer. At the indicated time point straight before the cells were harvested, the cells were visualized under an inverted phase-contrast microscope (Motic) to observe the cell morphological changes upon treatment, and photographs were taken.

### 2.7. Quantitative Real Time PCR (Q-PCR) Analysis of Proliferating Cell Nuclear Antigen (PCNA)

A total of 1 × 10^6^ Ca Ski cells were seeded into 60 mm culture dishes and incubated for 24 h for attachment. Cells were then treated without or with 20–100 *μ*M of MAA for 24 h. For time-course study, cells were treated with 60 *μ*M MAA for 0, 3, 6, 12, and 24 h. After specific treatment period, cells were harvested and total RNA was isolated using the RNAqueous-4PCR kit (Applied Biosystem) according to the manufacturer's directions. The RNA concentration was determined using spectrophotometry. The gene expression of PCNA was assessed by one-step SYBR Green relative real-time PCR (Rotor-Gene System, Qiagen) and normalized to GAPDH reference control amplifications. The primer sequences for PCNA and GAPDH were forward 5′-GCCTGCTGGGATATTAGCTC-3′, reverse 5′-CATACTGGTGAGGTTCACGC-3′ and forward 5′-CCAGGGCTGCTTTTAACTCTG-3′, reverse 5′-CGTTCTCAGCCTTGACGGTG-3′, respectively. The PCR amplification reactions were carried out in a total volume of 25 *μ*L for 30 cycles of 45 seconds at 95°C, 45 seconds at 56°C, and 120 seconds at 72°C. The mean fluorescence threshold value (C_T_) of each sample was obtained according to the manufacturer's guidelines and used to determine ΔC_T_ values where by ΔC_T_ = C_T target gene (PCNA)_ − C_T reference gene (GAPDH)_. The relative fold change in PCNA expression in the treated sample over the untreated control was calculated with the comparative ΔΔC_T_ method where ΔΔC_T_ = ΔC_T sample_ − ΔC_T untreated_ and was calculated using formula 2^−ΔΔCT^ [29].

### 2.8. Cell Cycle Analysis

The cell cycle distribution was assessed using propidium iodide (PI) staining [30]. Ca Ski cells were seeded in 60 mm culture dishes (1 × 10^6^ cells) and left 24 h for attachment. The cells were then treated without or with 60 *μ*M MAA for 12–72 h. After the designated treatment period, both adherent and floating cells were harvested and washed twice with PBS. Cell pellets were resuspended in 100 *μ*L of PBS and fixed with absolute ethanol and stored at −20°C for 24 h. Fixed cells were washed twice with PBS, and the cell pellets were incubated in a buffer containing 50 *μ*g/mL PI, 0.1% sodium citrate, 0.1% Triton-X-100, and 100 *μ*g/mL RNase A for 45 min in the dark at room temperature. The percentage of cells in the sub-G1, G1, S, and G2/M-phases of the cell cycle was then analyzed using a FACS Calibur flow cytometer (Beckton Dickinson). Data were acquired and analyzed using Cell Quest software (Becton Dickinson).

### 2.9. Data Analysis

All the results were presented as mean ± standard error (S.E.) of three experiments. Significant difference was analyzed by Student *t*-test. A *P*  value <0.05 was regarded as a significant difference from the corresponding control group.

## 3. Results and Discussion

### 3.1. Mollic Acid Arabinoside (MAA) and Mollic Acid Xyloside (MAX) Were Isolated from Ethyl Acetate Fraction of *L. indica* via MTT Bioassay-Guided Separation

**Figure 1:**
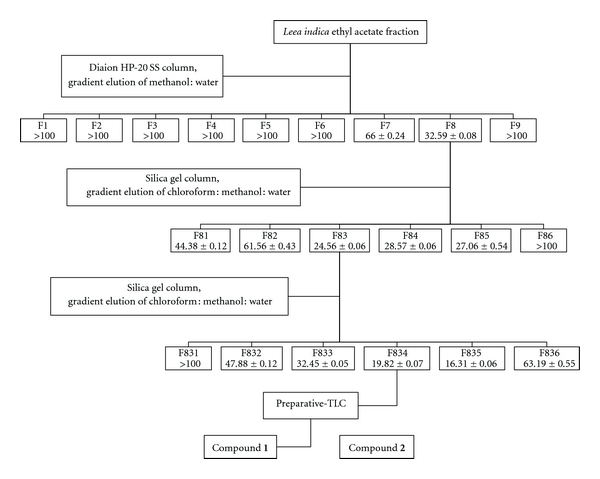
Flow chart of bioassay-guided isolation of cytotoxic compounds from the ethyl acetate fraction of* L. indica. *Each of the fractions was evaluated for its cytotoxic effect on Ca Ski cells using MTT assay. The IC_50_ values were means ± S.E. calculated from three experiments performed in triplicate.

Based on our previous study, the ethyl acetate fraction of *L. indica* demonstrated the strongest cytotoxic effect on Ca Ski cells [23]. Hence, it was subjected to MTT assay-guided isolation. The results were summarized in Figure 1. MTT test on the first 9 fractions (F1–F9) showed that Ca Ski cells were most susceptible to F8. Further separation of F8 yielded another 6 fractions (F81 to F86). Among the fractions, F83 was found to be the most effective. Subsequent fractionation of F83 yielded another 6 fractions (F831 to F836). The active F835 was subjected to prep-TLC and this led to the isolation of two compounds, compound **1 **and compound **2**.

**Figure 2:**
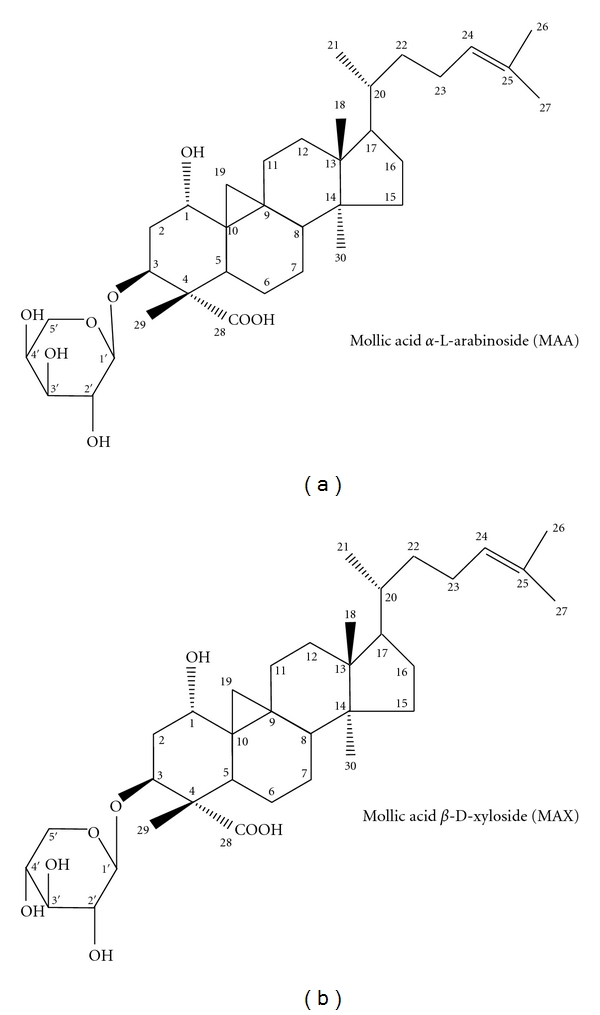
The chemical structures of the compounds isolated from *L. indica* via bioassay-guided approach.

**Figure 3:**
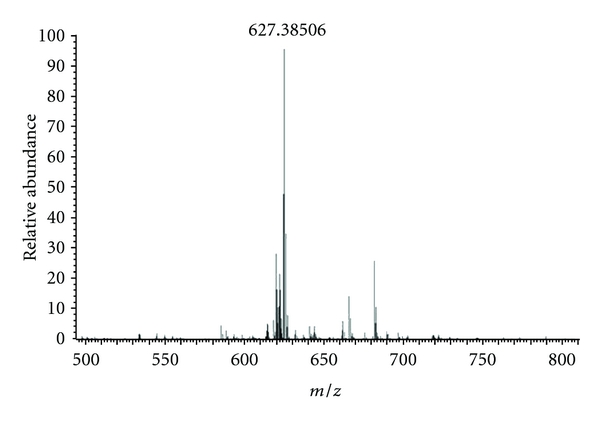
Positive ESI-MS spectrum of MAA or MAX. The ion at *m*/*z* = 627.39 represents the sodium adduct of that ion [M + Na]^+^.

They were identified as (**1**) mollic acid *α*-L-arabinoside (MAA) and (**2**) mollic acid *β*-D-xyloside (MAX) (Figure 2). They were isolated from *L. indica* for the first time. Their structures were confirmed by comparison of the obtained spectral data with the published literature data [31–33]. The structures were further confirmed by electrospray ionization mass spectrometry (ESI-MS), in a positive mode (Figure 3). The MS spectra showed the molecular ion peak at *m*/*z*: 627.3851, which corresponds to a molecular formula of C_35_H_56_O_8_.

Compound **1** was identified as 1*α*, 3*β*-dihydroxy-cycloart-24-ene-28-oic acid 3-O-[*α*-L-arabinopyranoside], C_35_H_56_O_8_. Positive ESI-MS *m*/*z*: 627.3851 [M+Na]^+^. ^1^H NMR (125 MHz, C_5_D_5_N): *δ* 0.47 (1 H, d, J = 4.0 Hz, H-19A), 0.75 (1 H, d, J = 4.0 Hz, H-19B), 0.85–1.66 (6 × CH_3_), 3.39–4.42 (arabinose protons; H-1 of aglycone), 5.01 (1 H, d, J = 6.6 Hz, H-1′ of arabinose), 5.21–5.5 (2 H, m, H-3 *α*, H-24). The ^13^C NMR data of compound **1** was shown in Table 1.

Compound **2** was identified as 1*α*, 3*β*-dihydroxy-cycloart-24-ene-28-oic acid 3-O-[*β*-D-xylopyranoside], C_35_H_56_O_8_. Positive ESI-MS *m*/*z*: 627.3851 [M+Na]^+^. ^1^H NMR (125 MHz, C_5_D_5_N): *δ* 0.44 (1 H, d, J = 4.0 Hz, H-19A), 0.72 (1 H, d, J = 4.0 Hz, H-19B), 0.91–1.68 (6 × CH_3_), 3.39–4.42 (xylose protons; H-1 of aglycone), 5.08 (1 H, d, J = 6.6 Hz, H-1′ of xylose), 5.20–5.48 (2 H, m, H-3 *α*, H-24). The ^13^C NMR data of compound **2** was shown in Table 2.

### 3.2. MAA and MAX Were Cytotoxic to Ca Ski Cells with Less Detrimental Effect on Normal Cells

**Figure 4:**
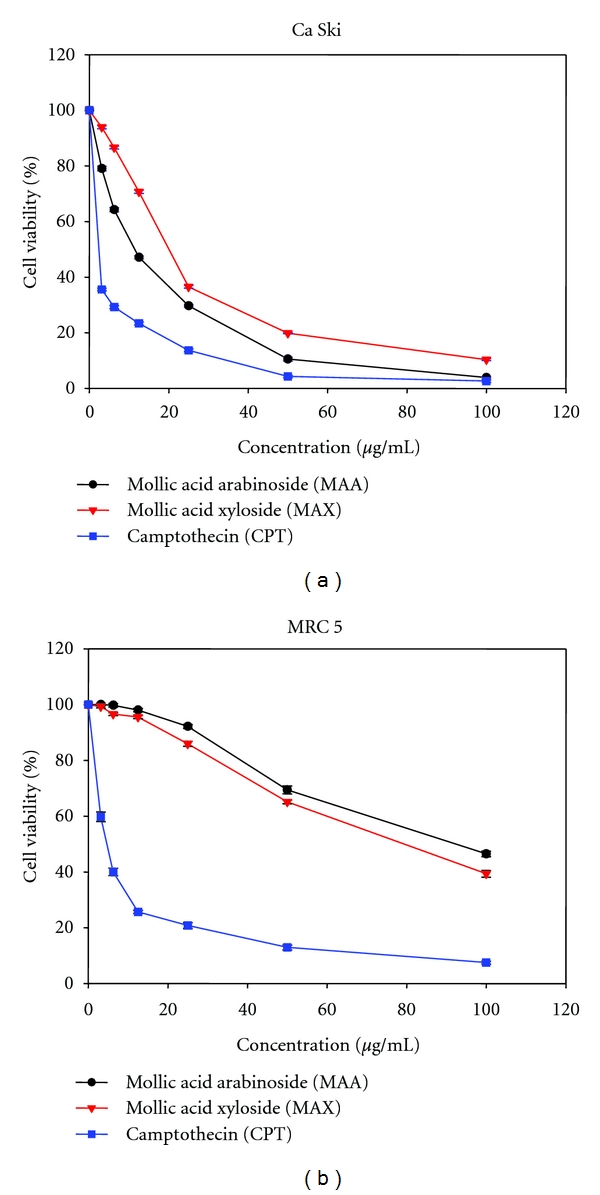
Effect of MAA and MAX on the cell viability of Ca Ski cervical cancer cells (a) and MRC 5 normal cells (b). Cells were left untreated or treated with increasing doses of MAA or MAX for 72 h. Camptothecin (CPT) was used as positive control. The cell viability was measured by MTT assay as described in method. The data were mean values ± S.E. of three different experiments.

**Figure 5:**
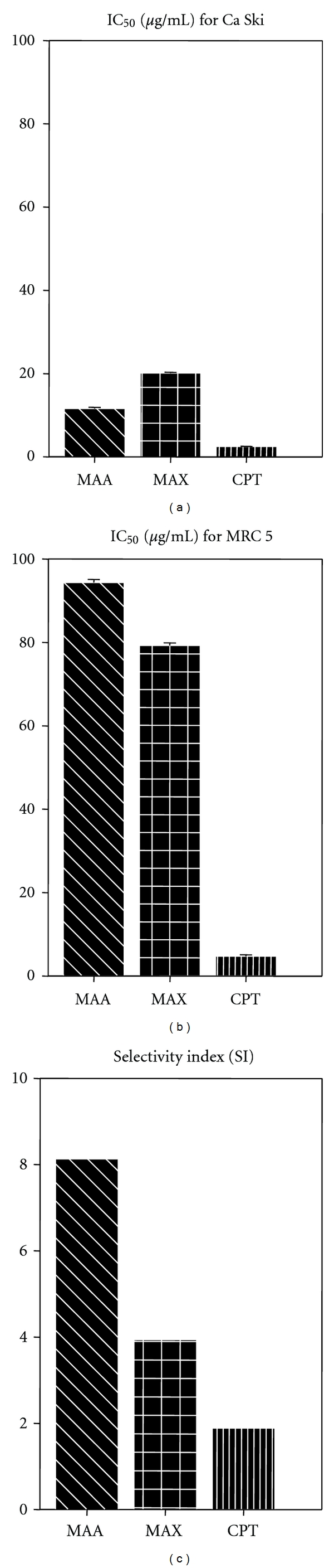
Bar charts showing IC_50_ and SI values of MAA, MAX, and camptothecin (CPT). IC_50_ (concentration that reduces cell viability to 50%) values were determined from the dose response curve generated by the MTT assay. SI (Selectivity index) values were determined by dividing the IC_50_ value of MRC 5 with the IC_50_ value of Ca Ski. Data for IC_50_ were means ± S.E. calculated from three experiments.

MAA and MAX were evaluated for their cytotoxic effect on Ca Ski cervical cancer cells and MRC 5 normal cells using MTT assay. A 72 h exposure to Ca Ski cells with MAA or MAX led to a significant dose-dependent reduction in cell viability. According to Figure 4, the decrease in cell viability ranged from 20–95% and 6–90% when the cells were treated with 3.125–100 *μ*g/mL of MAA and MAX, respectively. As shown in Figure 5, the IC_50_ values of MAA and MAX for Ca Ski cells were 11.60 ± 0.29 *μ*g/mL (19.21 *μ*M) and 20.13 ± 0.21 *μ*g/mL (33.33 *μ*M), respectively. When compared to Ca Ski cells, MAA and MAX were less cytotoxic to the normal cells, as revealed by the relatively higher IC_50_ values on MRC 5 (94.32 ± 0.75 *μ*g/mL for MAA and 79.25 ± 0.66 *μ*g/mL for MAX). In contrast, camptothecin (CPT) displayed comparable IC_50_ value on both Ca Ski and MRC 5 cells (2.51 ± 0.33 and 4.74 ± 0.33 *μ*g/mL). The small difference in IC_50_ value led to our deduction that CPT cannot differentiate between normal and cancer cells and killed both cells at almost the same efficiency. This lack of tumor-cell specificity was also reported by other scholar [34].

The primary goal of cancer chemotherapy is to target specifically at cancer cells but innocuous to normal cells. However, many anticancer drugs fail to meet this criterion, as they cannot discriminate between cancer and normal cells, which make them cytotoxic not only to cancer cells, but also to normal cells. Therefore, development of novel cancer chemotherapeutic agent with a higher potency and specificity against cancer cells is urgently needed. It is interesting to note that MAA and MAX exhibited approximately 20-fold and 17-fold higher IC_50_ values against MRC 5 when compared to CPT. Moreover, we also compared the cytotoxicity of MAA, MAX, and CPT based on their SI values. MAA was about 8 times more cytotoxic to Ca Ski cells compared to MRC 5, while MAX was about 4 times more cytotoxic to Ca Ski cells compared to MRC 5 (Figure 5).

### 3.3. MAA Caused Both Cytostatic and Cytocidal Effects on Ca Ski Cells

Since MAA exhibited higher SI and lower IC_50_ compared with MAX, it was selected for further investigations. To characterize the cytotoxic effect of MAA, we employed two cytotoxicity assays which measure different parameter of cell death, namely MTT (dye reduction) and TBE (dye exclusion) assays, which measure mitochondria metabolic death and cell membrane integrity, respectively. As mentioned earlier, we showed that MAA caused a conspicuous dose-dependent reduction of formazan formation in Ca Ski cells (Figure 4(a)). This indicated that the cytotoxic action was mediated via disruption of mitochondrial dehydrogenase system inside the cells.

**Figure 6:**
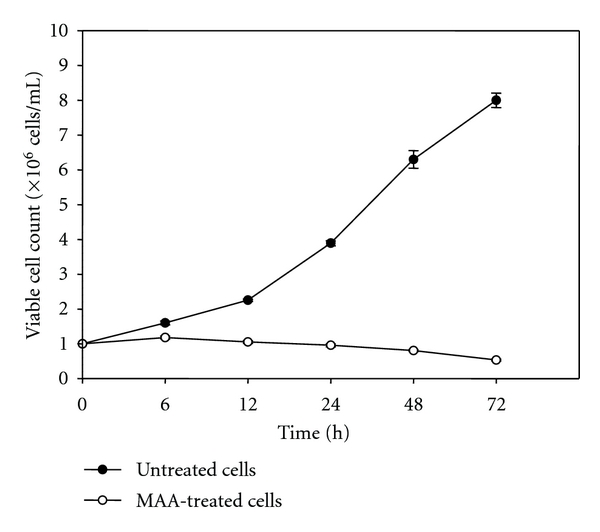
Effects of MAA on the viable cell count on Ca Ski cells. Cells were treated without or with 60 *μ*M of MAA for 6–72 h. At each time point, the viable cell numbers were counted using a hemocytometer as described in TBE assay in the methods. Each point represents means ± S.E. from three experiments.

**Figure 7:**
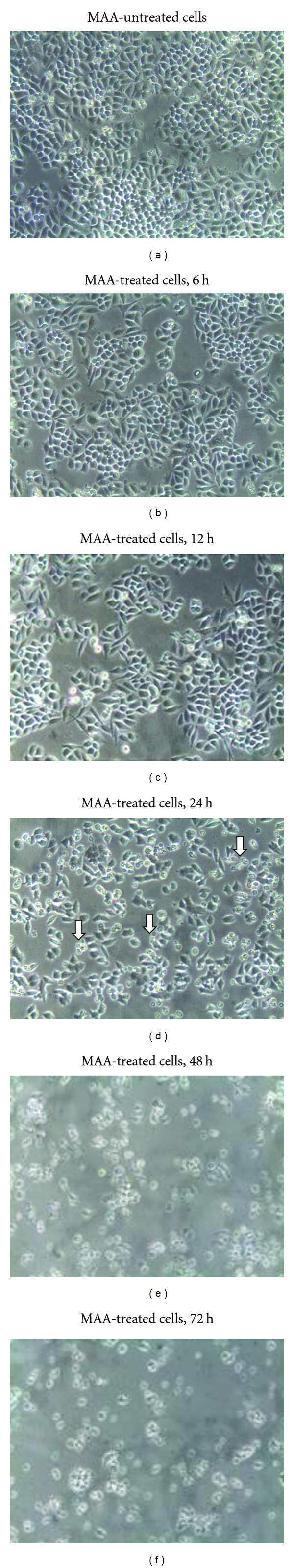
Effect of MAA on the morphological changes of Ca Ski cells. The MAA-untreated cells represented cells treated with vehicle DMSO (final 0.5% v/v) for 6 h. Cells were treated with 60 *μ*M of MAA for 6–72 h and visualized under microscope and photographed. Magnification: 100x. After treatment with MAA, cells showed progressive loss of normal elongated shape. Cells shrunk to smaller rounded cells (shown by the arrows) and detached from the surface.

The cytotoxic effect of MAA was further substantiated by the TBE assay. As shown in Figure 6, the control untreated cells proliferated faster compared to the treated cells, as demonstrated by the rapid exponential growth of the cells in the absence of MAA. In contrast, the cell proliferation was hindered when incubated with the presence of MAA. Treatment for 6 h modestly inhibited the cell proliferation, while prominent antiproliferative effect was observed at 12 h and 24 h. Prolong treatment resulted in decrease of cell number from the initial cell seeding density, indicating a more pronounced disruption of cell-membrane integrity (due to uptake of trypan blue). Simultaneously, we observed the cellular morphological changes during MAA treatment (Figure 7). The cells remained elongated in shape and attached at 6 and 12 h, while at 24–72 h, the cells progressively shrunk to smaller rounded shape and started to detach. During early hours (6–24 h) of treatment, cytostatic effect was evident, as shown by the inhibition of cell proliferation (antiproliferation) in the TBE assay. At prolong treatment, cytocidal effect became pronounce, as the cells died and detached from the surface. This was in agreement with the MTT 72 h end-point assay, in which the IC_50_ value obtained in the recovery assay is almost the same to the exposure assay. Hence, we can say that cytostatic and cytocidal effects were responsible for the cytotoxic effect of MAA in Ca Ski cells.

### 3.4. MAA Inhibited the Proliferation of Ca Ski Cells

**Figure 8:**
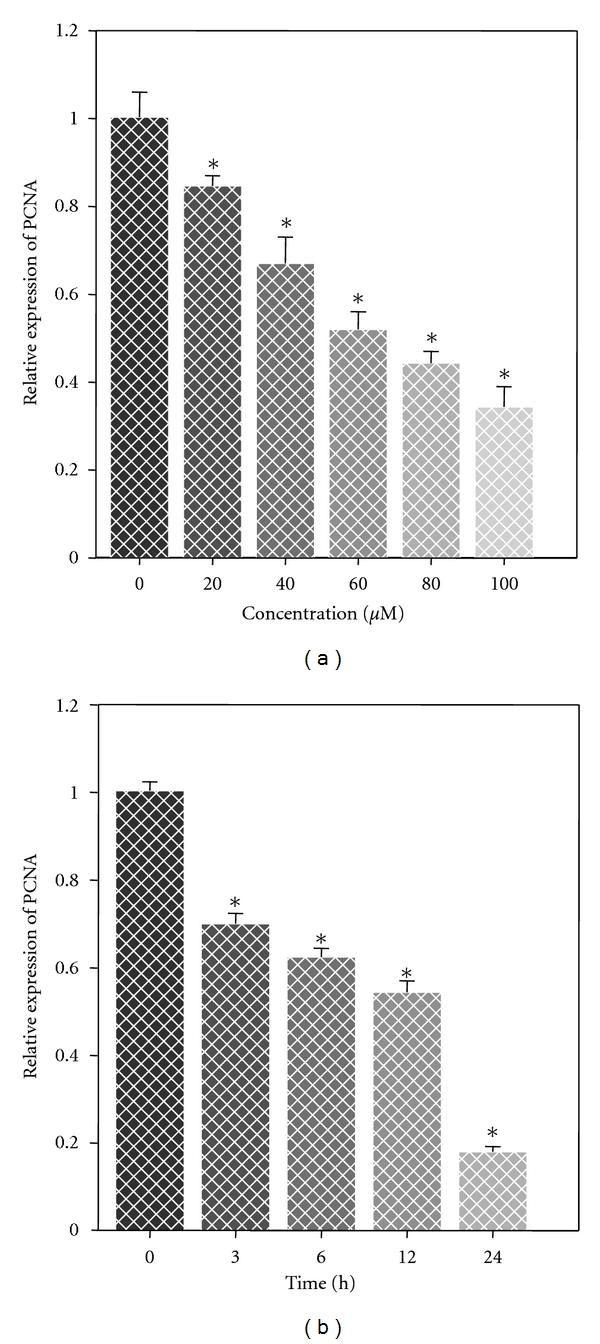
Effect of MAA on the PCNA expression in Ca Ski cells. Cells were incubated in the absence or presence of 20–100 *μ*M of MAA for 24 h. Cells were also incubated with 60 *μ*M of MAA for 0, 3, 6, 12, and 24 h. After indicated time, cells were collected; total RNA was extracted and Q-PCR was performed as described in methods. Results were expressed as relative expression of PCNA compared to the untreated control, normalized with GAPDH. Values were mean ± S.E. from three independent experiments.

Since we showed that MAA exerted cytostatic effect on Ca Ski cells, we next aimed to evaluate the antiproliferative effect of MAA. Previous reports have shown that proliferating cell nuclear antigen (PCNA) is greatly expressed in most of the proliferating cancer cells including cervical cancer [35, 36]. PCNA is a cell proliferation biomarker which plays a pivotal role in the decision of the life or death of the mammalian cells [37]. Hence, the effect of MAA on the expression level of PCNA was investigated. The cells were treated with MAA and the relative expression of PCNA was measured by Q-PCR. Results showed that MAA significantly decreased the expression of PCNA in a dose- and time-dependent manner (Figure 8). These data suggested that the antiproliferative effect of MAA could be attributed to the downregulation of PCNA expression.

### 3.5. MAA Induced Cell Cycle Arrest and Hypodiploid Cells

**Figure 9:**
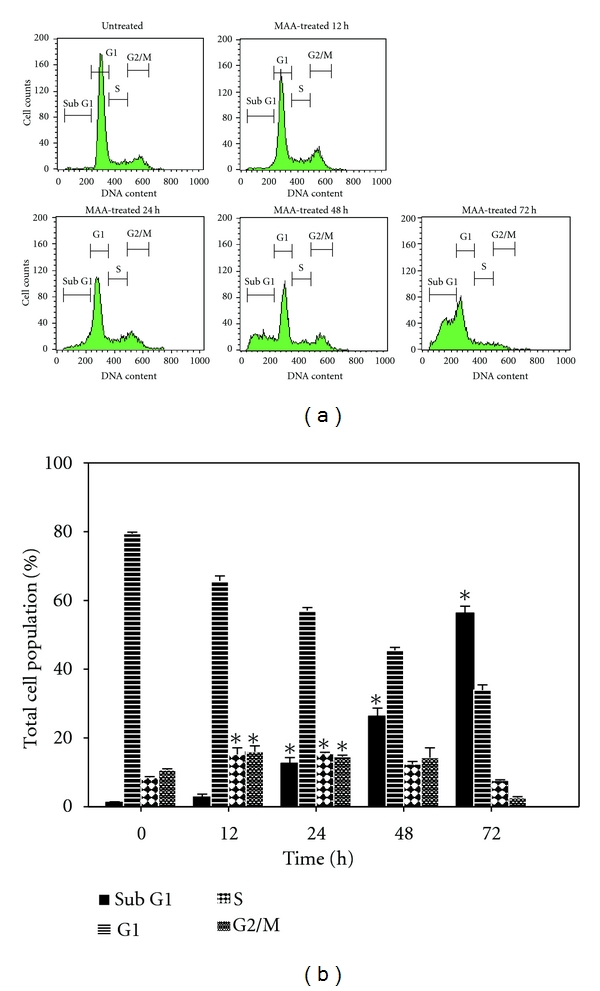
Effect of MAA on cell cycle phase distribution in Ca Ski cells. Cells were treated with 60 *μ*M MAA for 12–72 h. The untreated cells correspond to cells without MAA treatment at 12 h, and similar results were obtained at other incubation times. After treatment, cells were harvested, fixed, stained with PI, and analyzed by flow cytometry as described in methods. (a) Representative histograms showing cell cycle distribution. (b) Bar charts showing percentage of cells in sub-G1, G1, S, and G2/M phases of the cell. Results are mean values ± S.E. of three experiments. The percentage of the cell cycle phase in the treated cells was compared to the corresponding phase in the untreated control cells. Statistical significance is indicated by *P < 0.05.

Earlier, we have showed that the cytotoxicity of MAA in Ca Ski cells was derived from both cytostatic and cytocidal effects. Moreover, we also demonstrated that the cytostatic effect was due to antiproliferative effect, as reflected by the decrease of the proliferation marker, PCNA. Subsequently, we checked whether the antiproliferative effect was associated with any cell cycle phase-specific arrest. After treated with MAA for 12 and 24 h, the proportion of S-phase and G2/M-phase in the treated cells was significantly higher compared to the untreated cells. The perturbation of cell cycle progression, caused by the sustained accumulation of cells in the S and G2/M phases may be in part responsible for the cytostatic/antiproliferative effect of MAA. This was in agreement with the retardation of cell proliferation by MAA at 12 and 24 h in the TBE assay (Figure 6). In addition, after treated with MAA, cell cycle analysis showed a significant increase of hypodiploid cells (sub-G1) in a time-dependent manner. This was accompanied with a concomitant decrease of the cells in the G1 phase (Figure 9). Notably, the presence of hypodiploid cells started after 12 h of treatment and increased about 20-fold after 72 h. These hypodiploid cells are indicator of apoptotic cells [38]. However, other assays are needed to confirm the induction of apoptosis. Nevertheless, the present sub-G1 analysis served as a preliminary study on the apoptosis-inducing potential of MAA in Ca Ski cells.

The mollic acid glycosides (MAA and MAX) isolated from* L. indica *belong to the group called cycloartane triterpenoid glycoside. Recently, this group of compounds has received considerable attention for their cytotoxic potential [39, 40]. For mollic acid glycosides, their anticancer effects have not been explored yet based on the lack of scientific studies concerning their cytotoxic effect. In a previous study, mollic acid glycoside was suspected to be the compound responsible for the strong cytotoxic effect of *Combretum molle* on cancer cells [41]. However, no further study was conducted to verify the compound responsible for the cytotoxic action. In the present study, we firstly demonstrated that mollic acid glycosides exerted cytotoxic effect on cancer cells.

Therefore, our findings here warrant the need for further investigation on the anticancer potential of MAA, especially for cervical cancer. Elaborate studies to identify the mechanisms of action are in progress.

## 4. Conclusion

Conclusively, two cytotoxic cycloartane triterpenoid glycosides, namely mollic acid *α*-L-arabinoside (MAA) and mollic acid *β*-D-xyloside (MAX), were isolated form *L. indica* for the first time through bioassay-guided method. Preliminary studies showed that the cytotoxicity of MAA was associated with decrease of PCNA expression, cell cycle S and G2/M phases arrest, and induction of hypodiploid cells.

## Figures and Tables

**Table 1:** ^13^C NMR and DEPT 135 spectroscopic data of compound **1** (*δ* in p.p.m.; 75 MHz).

Carbon	Mollic acid *α*-L-arabinoside (C_5_D_5_N) [33]	Compound **1** (C_5_D_5_N)	
	^13^C	^13^C	DEPT
1	72.5	71.6	CH
2	37.7	36.8	CH_2_
3	81.5	80.6	CH
4	55.0	54.0	C
5	38.0	37.1	CH
6	23.3	22.4	CH_2_
7	26.0	25.4	CH_2_
8	48.4	47.5	CH
9	21.1	20.2	C
10	30.3	29.4	C
11	26.3	25.1	CH_2_
12	36.9	36.0	CH_2_
13	48.5	44.8	C
14	49.3	48.4	C
15	33.5	32.5	CH_2_
16	28.6	27.7	CH_2_
17	52.8	51.9	CH
18	18.5	17.6	CH_3_
19	29.8	28.9	CH_2_
20	36.4	35.5	CH
21	19.7	18.8	CH_3_
22	36.1	35.1	CH_2_
23	25.5	24.6	CH_2_
24	126.0	125.1	CH
25	131.0	130.1	C
26	26.0	25.1	CH_3_
27	18.0	17.0	CH_3_
28	180.2	179.6	C
29	10.5	9.7	CH_3_
30	18.7	17.8	CH_3_
1′	106.3	105.5	CH
2′	73.0	72.1	CH
3′	74.4	73.5	CH
4′	69.3	68.5	CH
5′	66.5	65.8	CH_2_

**Table 2:** ^13^C NMR and DEPT 135 spectroscopic data of compound **2** (*δ* in p.p.m.; 75 MHz).

Carbon	Mollic acid *β*-D-xyloside (C_5_D_5_N) [31]	Compound **2** (C_5_D_5_N)	
	^13^C	^13^C	DEPT
1	72.6	72.0	CH
2	37.5	37.0	CH_2_
3	81.4	81.3	CH
4	54.9	54.2	C
5	38.0	37.1	CH
6	23.0	22.4	CH_2_
7	28.7	29.0	CH_2_
8	48.4	47.6	CH
9	21.2	21.4	C
10	30.4	29.6	C
11	26.1	25.5	CH_2_
12	37.0	36.0	CH_2_
13	45.8	44.9	C
14	49.5	48.5	C
15	33.5	33.4	CH_2_
16	26.4	27.8	CH_2_
17	52.9	51.9	CH
18	18.8	17.8	CH_3_
19	30.0	29.4	CH_2_
20	36.4	35.8	CH
21	18.8	17.7	CH_3_
22	36.5	35.5	CH_2_
23	25.5	24.6	CH_2_
24	125.2	125.2	CH
25	131.0	130.2	C
26	25.7	25.2	CH_3_
27	18.0	17.9	CH_3_
28	180.2	181.4	C
29	10.5	10.1	CH_3_
30	19.7	18.8	CH_3_
1′	106.5	105.6	CH
2′	75.5	74.7	CH
3′	78.1	77.2	CH
4′	71.2	70.5	CH
5′	67.1	66.4	CH_2_
